# The Absence of Evidence is Evidence of Non-Sense: Cross-Sectional Study on the Quality of Psoriasis-Related Videos on YouTube and Their Reception by Health Seekers

**DOI:** 10.2196/11935

**Published:** 2019-01-16

**Authors:** Simon M Mueller, Pierre Jungo, Lucian Cajacob, Simon Schwegler, Peter Itin, Oliver Brandt

**Affiliations:** 1 Department of Dermatology University Hospital Basel Basel Switzerland

**Keywords:** psoriasis, YouTube videos, layperson, poor quality, misleading information, dangerous content

## Abstract

**Background:**

Approximately 80% of internet users access health information online and patients with chronic illnesses especially rely on internet-based resources. YouTube ranks second among the most accessed websites worldwide and hosts an increasing number of videos with medical information. However, their quality is sometimes unscientific, misleading, or even harmful.

**Objective:**

As little is known about YouTube as a source of information on psoriasis, we aimed to investigate the quality of psoriasis-related videos and, if necessary, point out strategies for their improvement.

**Methods:**

The quality of the 100 most viewed psoriasis-related videos was assessed using the DISCERN instrument and the Global Quality Scale (GQS) by categorizing the videos into useful, misleading, and dangerous and by evaluating the reception of the videos by users.

**Results:**

Evaluation of the videos exhibited a total of 117,221,391 views and a total duration of 10:28 hour. The majority of clips contained anecdotal personal experiences with complementary and alternative psoriasis treatments, topical treatments, and nutrition and diets being the most frequently addressed topics. While advertisements accounted for 26.0% (26/100) of the videos, evidence-based health information amounted to only 20.0% (20/100); 32.0% (32/100) of the videos were classified as useful, 52.0% (52/100) as misleading, and 11.0% (11/100) as even dangerous. The quality of the videos evaluated by DISCERN and GQS was generally low (1.87 and 1.95, respectively, on a 1 to 5 scale with 5 being the maximum). Moreover, we found that viewers rated poor-quality videos better than higher quality videos.

**Conclusions:**

Our in-depth study demonstrates that nearly two-thirds of the psoriasis-related videos we analyzed disseminate misleading or even dangerous content. Subjective anecdotal and unscientific content is disproportionately overrepresented and poor-quality videos are predominantly rated positively by users, while higher quality video clips receive less positive ratings. Strategies by professional dermatological organizations are urgently needed to improve the quality of information on psoriasis on YouTube and other social media.

## Introduction

Social media has become increasingly important in the context of health care [1,2], and 80% of internet users access health information online, especially patients with chronic illnesses who rely on internet-based resources [2-5]. In particular, videos are powerful tools to disseminate medical information [2,3,6]. YouTube, an open access video-sharing platform, ranks second among the most accessed websites worldwide and hosts an increasing number of videos with medical information [7-9]. YouTube counts 5 billion visits per day [7,9] and 1 billion hours watched daily [8]. Distribution of medical information to such a huge audience offers invaluable opportunities but also dangers as the quality of unfiltered information posted is often unscientific, misleading, or even harmful [2,5,10-17]. While the role and quality of YouTube videos have already been investigated in various medical specialties, only little is known about this topic in dermatology. A descriptive analysis of 100 videos covering dermatology, sun protection, skin cancer, skin cancer awareness, and skin conditions yielded over 47 million views reflecting the high demand for dermatological information posted on YouTube [18]. Psoriasis is a hot topic in social media with psoriasis foundations and associations being among the most popular dermatology-related organizations on Facebook, Twitter, and LinkedIn [19]. The benefits of these social networks providing psoriasis patients with educational information has been reported [20]. Facebook and Instagram recently attracted public attention after removing psoriasis images that they categorized as content that may not meet community guidelines [21]. Two previous publications showed that YouTube is heavily accessed as a source of information on psoriasis [22,23]. They pointed to a dominance of privately posted videos and a lack of evidence-based medical information from trustworthy institutions. However, as little is known about the scientific quality of these videos, we sought to analyze psoriasis-related videos using the Global Quality Scale (GQS) and the DISCERN tool (note that DISCERN is not an acronym but the name of the instrument) and by classifying the videos as useful, misleading, or dangerous. Furthermore, since we hypothesized that it might be difficult for laypersons to adequately judge the quality of videos, we correlated the quality of the videos with the numbers of likes and dislikes to assess viewers’ ability to recognize high- and low-quality content. In addition, we analyzed the topics covered in the videos and their license types and upload sources in order to obtain a comprehensive picture of psoriasis-related YouTube videos. In summary, the objectives of this study were as follows:

- Identify upload sources, common topics, and YouTube categories of the 100 most-viewed videos
- Investigate the quality of YouTube videos as a source of information on psoriasis by applying two different score instruments
- Correlate viewers’ ratings with our quality assessments
- Point out strategies for interventions that increase the quality of psoriasis video clips and medical content in general uploaded to YouTube and other social media platforms

## Methods

### Data Collection

In this cross-sectional study, YouTube was searched on July 27, 2017, using the term psoriasis and the filter settings English UK (language) and United Kingdom (country). Subsequently, videos were sorted by their view count. Non-English videos or channels were excluded until the top 100 videos in English were displayed (Multimedia Appendix 1). We decided to limit our analysis to the first 100 clips since this is a common and accepted procedure when investigating YouTube videos [13,16,18,24,25]. Furthermore, in our study, the first 100 videos achieved a total of 73 million views, whereby the clip in the hundredth place only achieved about 40,000 views. This suggests that videos ranked below the first 100 have only a minor impact on the outcomes.

After collecting qualitative and quantitative data (duration, upload data, source, likes/dislikes, category, license type), overall quality of the videos was assessed by 5 experienced dermatologists using two assessment tools [26]. To optimize interrater agreement on the videos, the dermatologists attended training sessions to get familiar with quality assessments and the rating policy and criteria.

### Creation of Content Categories

In a first step, topics of the video clips were collected. If a video covered more than one topic, each topic was listed separately. The content was subsequently categorized according to commonalities and by topics and/or categories discussed in two previous YouTube studies on psoriasis [22,23]. Unlike other studies that only used titles to categorize topics, we exclusively considered content for categorization, as the title often does not reflect the actual content of the clip.

### Scoring and Classification of Videos

The GQS, which is based on a 5-point scale, was developed in 2012 by Singh et al [16] for the evaluation of YouTube videos and has since been applied in numerous studies. The score measures the quality and flow of information and the value of an information source for medical laymen (Multimedia Appendix 2, Table A).

The DISCERN instrument is used to measure the quality of health information about treatment choices provided in video clips [27]. Originally developed for the standardized assessment of written medical information, the DISCERN tool consists of 15 key questions and an evaluation of overall quality with which the reliability (questions 1 to 8), quality of information (questions 9 to 15) and overall quality of a publication (question 16) can be assessed by assigning 1 to 5 points per question (Multimedia Appendix 2, Table B). For both tools, the higher the total value, the higher the quality of the video clip.

In addition, videos were classified into useful, misleading, or dangerous and categorized by topic or content, presence and profession of a presenter (to be seen in the video, health or nonhealth professional), and upload sources. In case of differing assessments by the analyzing dermatologists, the corresponding video was reassessed by the principal investigator (SM).

### Statistical Analyses

Descriptive statistics and Spearman correlation coefficients to calculate the number of likes and dislikes with the values of DISCERN and GQS, respectively, were performed using SPSS Statistics version 22.0 (IBM Corp). To assess the interrater reliability, the Cohen kappa coefficients and intraclass correlation coefficients were calculated.

## Results

### View Count, Duration, Upload Sources, Categories, and Topics

The 100 videos had a total of 117,221,391 views and a total duration of 10:28 hours (mean duration per video 6:17 [SD 6:39] minutes). The two most viewed videos accounted for 93,736,280 views (79.96% of the total) and were pharmaceutical advertising with a Creative Commons license and likes, dislikes, and comment functions disabled. This kind of license authorizes users not only to download the video but also to use the entire clip or parts thereof for their own video clip productions. The other 98 videos had a Standard YouTube License, which allows the use of the clips only after prior permission of the author.

The majority (65/100, 65.0%) of the videos were uploaded from the United States. The most frequent category was People & Blogs (36/100, 36.0%), a diverse mix of content and the most famous category on YouTube [28], while 27.0% (27/100) appeared in the category Education, 21.0% (21/100) in Howto & Style and only 7.0% (7/100) in Science and Technology.

**Figure 1:**
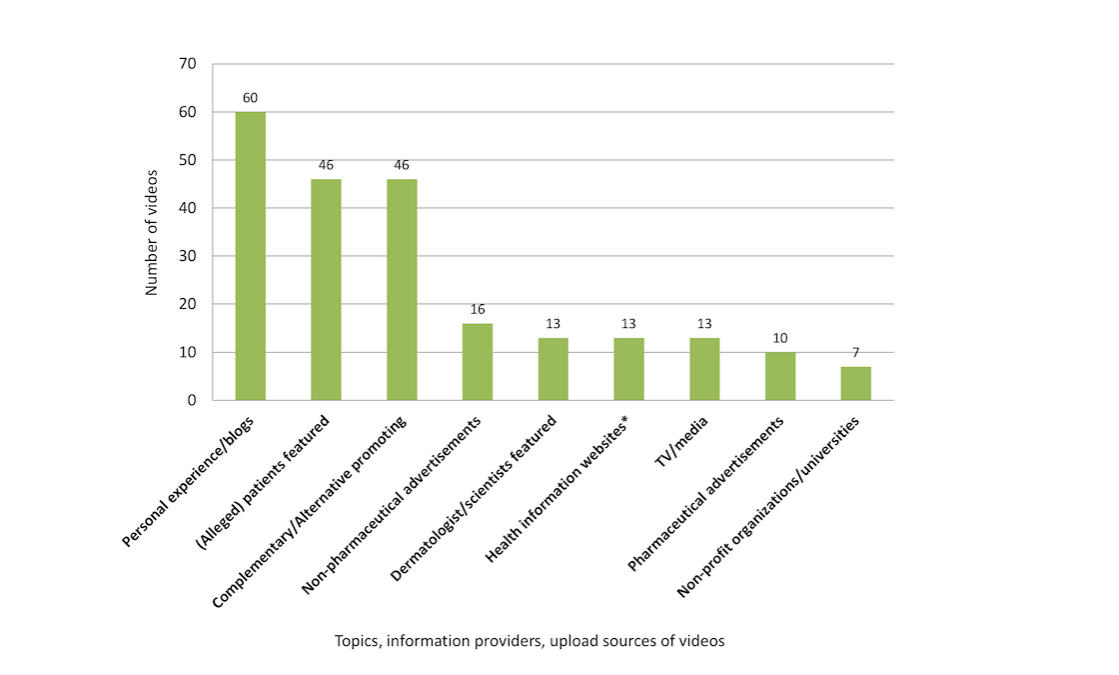
Distribution of topics, information providers and upload sources (multiple categories may apply to one video); *including websites from psoriasis associations.

**Figure 2:**
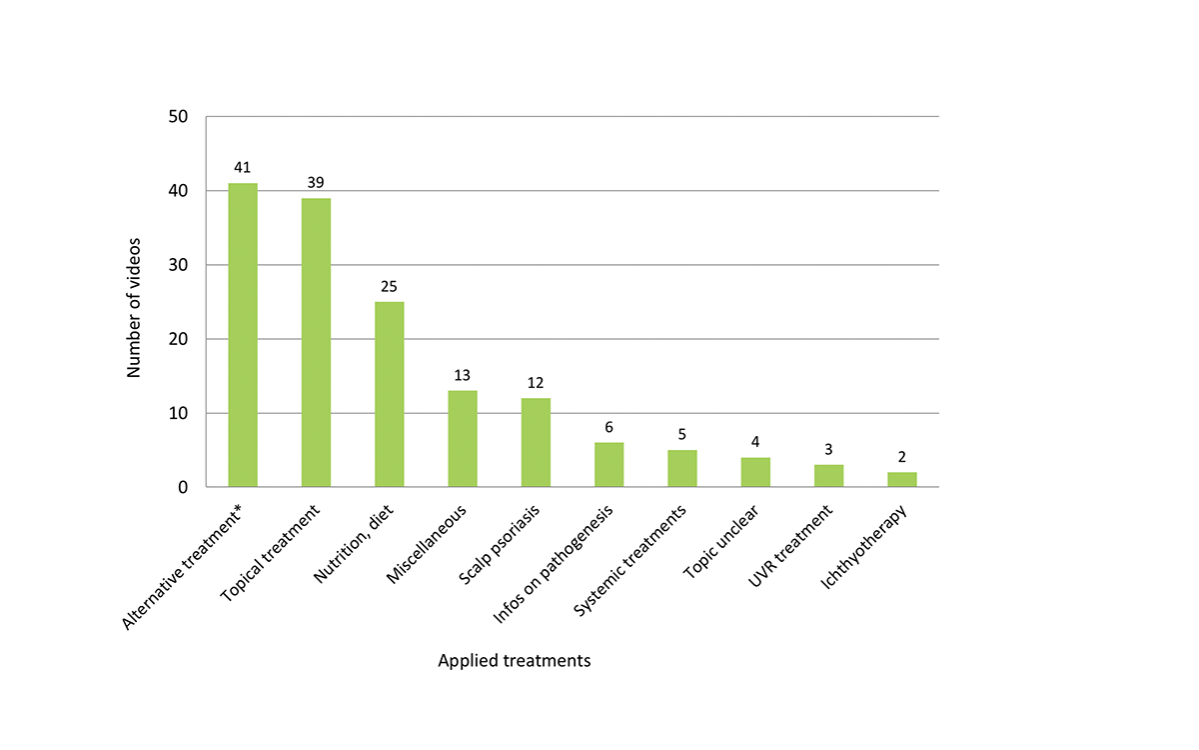
Topics presented in the videos (Note: a video clip can cover more than one topic; *alternative treatment includes complementary treatment; UVR: ultraviolet radiation).

**Figure 3:**
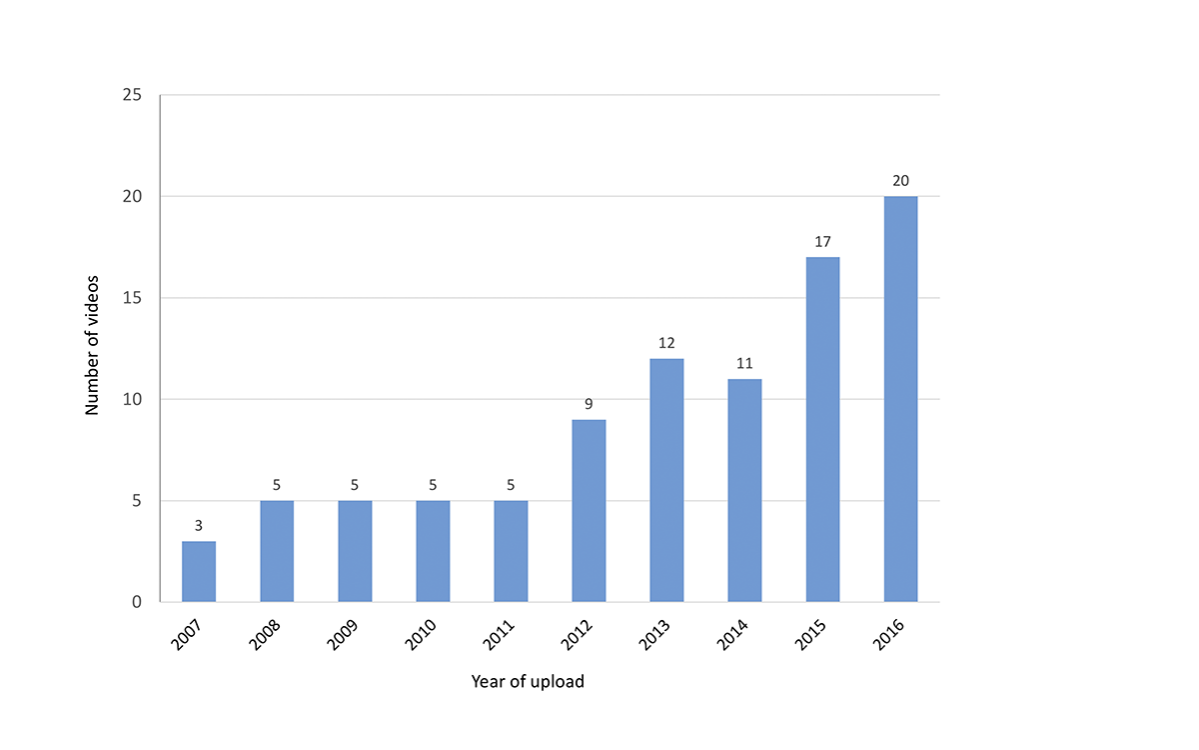
Distribution of the uploaded videos over the period 2007-2016 (n=100).

Most videos contained anecdotal, personal, unscientific, or commercial information on psoriasis (Figure 1). While pharmaceutical and nonpharmaceutical advertisements made up 26.0% (26/100) of the video clips, evidence-based health information posted by professional individuals and institutions amounted to only 20.0% (20/100). Complementary and alternative psoriasis treatments (41/100, 41.0%), topical treatments (39/100, 39.0%), and nutrition/diets (25/100, 25.0%) were the most often addressed topics (Figure 2). Video clips were uploaded between September 2007 and June 2016, with numbers increasing from 2012 on (Figure 3). In some videos, the comment function was disabled and the YouTube statistics were not declared.

**Figure 4:**
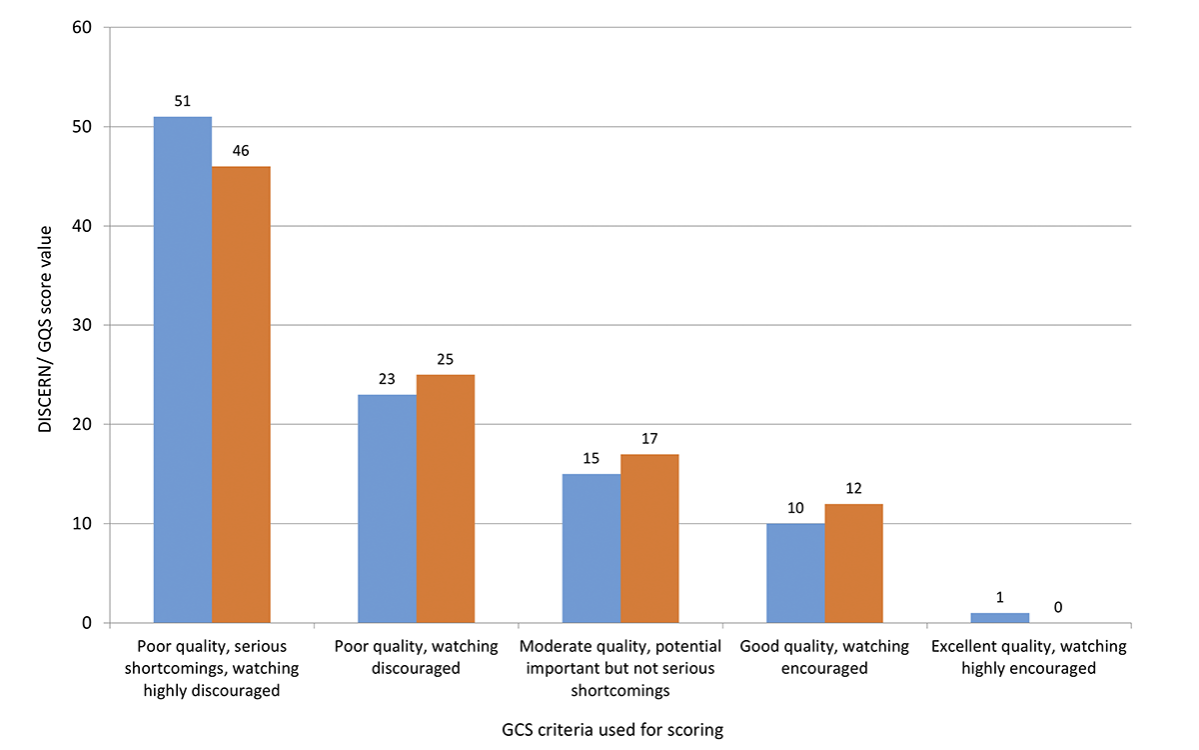
Comparison of quality assessments of the videos (n=100) performed with the DISCERN instrument (blue bars) and the Global Quality Scale (orange bars).

### Quality Assessments and Correlation With Likes and Dislikes

Of the videos, 32.0% (32/100) were classified as useful and 63.0% (63/100) as misleading—of these, 17.5% (11/63) were even considered dangerous because of potential mechanical or chemical injury or harmful recommendations regarding sun exposure or diets; 5% (5/100) of the videos were neither useful nor misleading.

With a value of .74, the kappa statistic revealed a good level of agreement among the raters.

In terms of the view count, we excluded the 2 most viewed videos from further analyses as they were pharmaceutical advertisements, accounting for 79.96% (93,736,280/ 117,221,391) of all views. The misleading videos (63/100) garnered 18,387,077 views including 4,611,126 views of videos with potentially dangerous content (11/63). Useful videos (32/100) had 5,098,034 views resulting in a ratio of 3.61 (18,387,077:5,098,034) misleading to useful videos.

The ratings using the DISCERN and GQS scores were consistent, yielding the categorizations shown in Figure 4. The quality of the videos, expressed by the mean overall DISCERN and GQS rating scores, was generally low (1.87 [SD 1.07] and 1.95 [SD 1.06], respectively) on a 1 to 5 scale with 5 being the maximum. Detailed analysis of the DISCERN values revealed that the major shortcomings were lack of information about the evidence and source of the posted information, areas of uncertainty and risks of the praised therapy, and missing recommendations for shared decision making or links to additional sources of information (see Multimedia Appendix 3).

The intraclass correlation coefficients calculated for the DISCERN and GQS were .81 and .78, respectively, indicating a high level of agreement between the assessors.

The videos received 113,147 likes and 9260 dislikes yielding a like to dislike ratio of 12.4 (113,147:9260). In 7 videos, the like/dislike function was disabled. When correlating the viewer ratings with our quality assessments, we found a negative correlation between the number of likes and the DISCERN mean values (Spearman correlation coefficient ρ=–0.23, *P*=.24) and a negative correlation between the number of dislikes and the DISCERN and GQS mean values (ρ=–0.34, *P*=.001, and ρ=–0.37, *P*<.001, respectively), meaning that viewers rated poor quality better than higher quality videos.

## Discussion

### Principal Findings

Psoriasis patients are avid users of social media, including YouTube, as a source of information on their disease [19-23,29]. However, little is known about the upload sources, topics, and particularly the scientific quality of these YouTube videos. Moreover, it is unknown whether viewer ratings correlate with the quality of the medical information posted.

This study found that the majority of video clips contained anecdotal personal experiences mainly addressing topics such as alternative treatment options for psoriasis and putative benefits of diets. Alarmingly, more than half of the videos spread misleading and about 1 in 10 even dangerous information and recommendations. Furthermore, the quality of the video clips was rather low, and the fact that viewers rated poor quality better than higher quality videos indicates that the majority of health seekers are not capable to recognize low quality medical information in videos as such.

### Misleading Information

We found that nearly two-thirds of the top 100 psoriasis-related YouTube videos disseminate misleading information. While this proportion of misleading content is in line with previous nondermatological studies [14,15,17,30,31], no direct comparisons in terms of psoriasis-related studies are currently available. Qi et al [23] have analyzed 47 psoriasis videos with a total of 2 million views but only distinguished between useful (18 videos) and misleading (10 videos), which makes a direct comparison with our results impossible. Lenczowski et al [22], on the other hand, did not judge the quality of the psoriasis videos they investigated.

It is unclear why the two pharmaceutical company videos account for almost 80% (93,736,280) of the more than 117 million visitors to the top 100. As the statistical information provided by YouTube did not allow us to determine whether the videos accessed were actually or completely viewed and when they were left, the significance of this figure is limited. It is, however, conceivable that at least some of the visits were due to the Creative Commons License, which, as mentioned above, allows the use of YouTube videos for personal video clip productions.

### Potentially Dangerous Content

A total of 11% of the videos we analyzed contained potentially dangerous content. For example, psoriasis patients were encouraged to remove their plaques using a knife blade, glue, Brazilian waxing, and apple cider vinegar. In addition, sunbathing without reference to sun protection, unnecessary diets (eg, avoidance of dairy or gluten), and the use of the one and only miracle cure were praised in such videos. This advice was frequently posted by patients reporting a personal negative long-term experience with conventional medicine who eventually found salvation in alternative treatments. It has been reported that patients with moderate to severe psoriasis are more apt to rely on psoriasis user–generated content in social media than their counterparts suffering from milder forms [29]. Therefore, it can be assumed that patients with more severe forms may be more prone to follow dubious advice and thus have an increased risk for undertreatment of skin and joint inflammations favoring the progressive psoriatic march.

### Low Percentage of Good or Excellent Quality Videos

Interestingly, the main topics published in psoriasis-related videos revolve around complementary and alternative psoriasis treatments, (homemade) current therapies, and nutrition and diet topics that allow patients to take measures to improve their skin condition without consulting a physician or health care professional. According to the results of the GQS and DISCERN tools, only 11% and 12% of the videos, respectively, were of good or excellent quality with unbiased, evidence-based or at least science-based information. This and our analyses of the uploaded sources indicate, in accordance with Lenczowski et al [22], that health care organizations, universities, and dermatologists are clearly underrepresented on YouTube in the context of psoriasis. The issue of a lack in high-quality information seems to be exacerbated by the negative correlation we found between the quality and the number of likes: high-quality videos are not as popular as low-quality videos. This finding is in line with Qi et al [23], who reported that useful psoriasis videos had fewer likes than misleading ones. The trend of nonuseful videos being more popular than useful ones has also been reported by nondermatological studies [11,13,31]. It remains unclear why viewers appear to like low-quality videos more than high-quality ones, and we can only speculate about the reasons. It is conceivable that (1) they just do not recognize high quality, (2) high-quality videos are too complex and less entertaining, or (3) viewers are intentionally looking for unconventional content diverging from established medical recommendations. The latter possibility may be supported by the observation of Lenczowski et al [22], which suggests that unconventional videos receive more views and likes than traditional medical videos. Remarkably, not only did a high number of likes correlate with low quality, a low number of positive ratings correlated with high-quality video clips. Exploration of viewer comments could help to elucidate the relationship between the likes and dislikes and the quality of the content.

These findings raise question about why certain viewers are so drawn to low-quality videos and how best to deal with this phenomenon to bring about change. To answer this, it is first of all helpful to gain knowledge of the characteristics of the typical psoriasis health seeker [22,32]. Previous studies demonstrated that health seekers in general have limited skills in searching and evaluating medical content on the internet and rarely call up results that appear beyond the second results page [33,34]. Websites without commercial advertising using medical terms enjoy more trust among the majority of users and are not left as quickly as those that do not meet these criteria [33,35]. Moreover, the majority of individuals searching the internet for medical information feel confident when advice matches what they already know or think they know about the subject and when similar information about it is available on more than one site [32,35]. Furthermore, we found that more than one-third of the videos we analyzed were uploaded to the popular YouTube category People & Blogs, which allows conclusions to be drawn about the claims and ideas of those who address the public with their supposed knowledge. From these findings, some of the necessary steps toward a better information policy for psoriasis patients in the social media can be derived.

### Possible Interventions

First, it is urgently necessary that dermatology associations, psoriasis self-help organizations, etc, provide medically accurate, high-quality, and easy-to-understand information—including videos—for laymen dealing not only with pathophysiology, clinical manifestations, and evidence-based therapeutic options for psoriasis but also with non–evidence-based treatments and their inefficacy and potential hazards.

Second, it is important to keep in mind that YouTube videos reach a large audience, a fact that the World Health Organization has recognized; it explicitly recommends the use of this platform in its strategic communications framework [36]. In our analysis, YouTube provided more than 5 million viewers with partly useful information on psoriasis. However, 3.6 times more viewers watched videos with misleading information indicating that YouTube may be a double-edged sword. Most viewers are likely to watch several videos including both misleading and useful ones. As it is conceivable that misconceptions may be corrected by useful information, it is crucial that the number of videos on psoriasis posted by professional health organizations is sufficient to neutralize the misleading ones. Quality assurance measures are essential to achieve this.

To our knowledge, this is the first study to include the DISCERN tool in the context of dermatology-related YouTube videos. The mean score gained from the overall rating of the DISCERN tool (question number 16) was very similar to the GQS score (both approximately 1.9). However, as the DISCERN scoring system provides important additional information, we believe that this tool is better suited for quality assessment of videos and should therefore also be used to evaluate films from professional health organizations before they are posted.

Third, as the majority of health seekers rely on the first 10 results from the search engine, it is important to ensure that websites, videos, etc, of professional health care providers appear on the first 2 result pages [33]. To achieve this, search engine optimization can be performed by using as many keywords that health seekers regularly use in their research as possible in domains and meta elements (metatags), headlines, and in the bodies of the text [34].

Furthermore, cooperation between social media and search engine providers and dermatology associations and psoriasis self-help organizations would enable the positioning of medically accurate information in a prominent location on the results page and easier access to corresponding websites and videos. Such an approach had already proved successful in 2003 during the SARS epidemic, when the internet company Google ensured that the World Health Organization and Centers of Disease Control and Prevention websites were displayed at the top of the first results page in its search engine [32].

Finally, one could also think about subjecting websites and video clips with medical content to standardized quality control or setting minimum standards through government regulations. However, due to the amount of media posted daily and the philosophy of the YouTube platform, this seems hardly feasible.

### Strengths and Limitations

Despite their unmistakable strengths, such as the comprehensive analyses of a high number of videos and the application of two different scoring tools (GQS and DISCERN), there are some limitations to our study. Although we performed comprehensive analyses, we neither evaluated the comments posted by viewers nor did we investigate potential associations between the duration of YouTube videos and their quality, the number of likes and dislikes they received, etc, which might have allowed us, among other things, to make statements about preferences of viewers and optimal durations of video clips.

### Conclusion

Our study demonstrates and confirms the results of others that the vast majority of psoriasis videos presented on YouTube contain misleading and sometimes potentially harmful information about this disease. Moreover, our findings suggest that a large number of users looking for clips on psoriasis on YouTube are not only unable to distinguish between medically accurate and inaccurate information but even tend to rate videos of inferior quality better than videos of higher quality.

According to our findings and in agreement with the five previous dermatological YouTube studies [18,22,23], it is crucial that dermatology associations and health facilities identify the motives of the users for this behavior. This would enable them, together with operators of social media platforms and state institutions, to develop strategies aimed at improving the quality of the information provided on YouTube and other social media platforms. The evidence-based material should be created in such a way that it can easily be found by search engines and appear on the first 2 results pages.

## Appendix

Multimedia Appendix 1 Links to YouTube videos.

Multimedia Appendix 2 Scores used for quality evaluation of video clips.

Multimedia Appendix 3 Rating of the 15 items of the DISCERN tool on a scale from 1 to 5 (with 5 being the maximum).

## Abbreviations

GQS: Global Quality Score
UVR: ultraviolet radiatio
