# Supplementary material for: The Absence of Evidence is Evidence of Non-Sense: Cross-Sectional Study on the Quality of Psoriasis-Related Videos on YouTube and Their Reception by Health Seekers
Source: J Med Internet Res. 2019 Jan 16;21(1):e11935. doi: 10.2196/11935 (PMC6357908; doi:10.2196/11935)
Supplement: Multimedia Appendix 2 [file jmir_v21i1e11935_app2.pdf]

Multimedia Appendix 2. Scores used for quality evaluation of video clips.

Table A. Global Quality Score used for evaluation of video clips.

Item characteristics	Points
Poor quality; poor flow of the video; most information missing; not at all useful for patients	1
Generally poor quality and poor flow; some information listed, but many important topics missing; of very limited use to patients	2
Moderate quality; suboptimal flow; some important information adequately discussed, but other information poorly discussed; somewhat useful for patients	3
Good quality and generally good flow; most of the relevant information listed, but some topics not covered; useful for patients	4
Excellent quality and flow; very useful for patients	5

Table B. DISCERN instrument for evaluating health information of online videos (adapted from Charnock [24]).

Questions	Points
Are the aims clear?	1-5
Does the video clip achieve its aims?	1-5
Is it relevant?	1-5
Is it clear what sources of information were used to compile the video?	1-5
Is it clear when the information used or reported in the video was produced?	1-5
Is the content balanced and unbiased?	1-5
Does the video provide details of additional sources of support and information?	1-5
Does it refer to areas of uncertainty?	1-5
Does it describe how each treatment works?	1-5
Does it describe the benefits of each treatment?	1-5
Does it describe the risks of each treatment?	1-5
Does it describe what would happen if no treatment was used?	1-5
Does it describe how the treatment choices affect overall quality of life?	1-5
Is it clear that there may be more than one possible treatment choice?	1-5
Does it provide support for shared decision making?	1-5
Overall rating of the video based on the answers to all of the above questions	1-5
