# Supplementary material for: The Absence of Evidence is Evidence of Non-Sense: Cross-Sectional Study on the Quality of Psoriasis-Related Videos on YouTube and Their Reception by Health Seekers
Source: J Med Internet Res. 2019 Jan 16;21(1):e11935. doi: 10.2196/11935 (PMC6357908; doi:10.2196/11935)
Supplement: Multimedia Appendix 3 [file jmir_v21i1e11935_app3.pdf]

Appendix 2

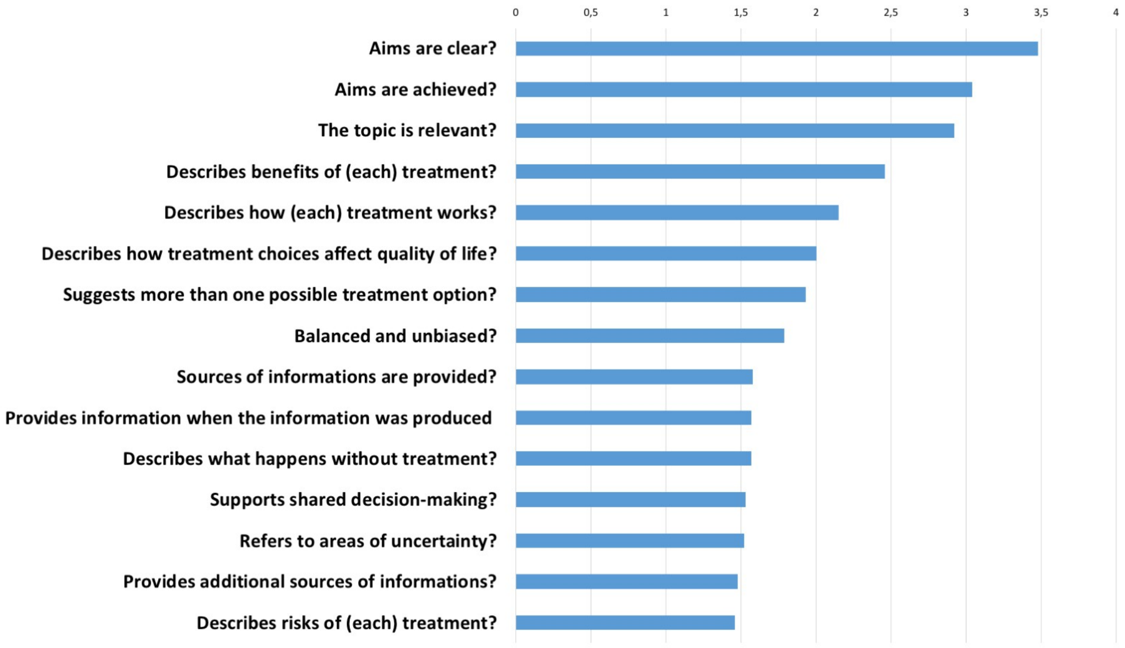

Appendix 2

Rating of the 15 items of the DISCERN tool on a scale from 0 to 5 (with 5 being the maximum).
